# Histological *Helicobacter pylori* Density Might Not be Associated With the Severity of Neutrophilic Inflammatory Activity

**DOI:** 10.1002/deo2.70356

**Published:** 2026-05-27

**Authors:** Guochun Lou, Zhengchen Yu, YongWei Wang, BingQiang Xu, Qin Du, Jun Ye

**Affiliations:** ^1^ Department of Gastroenterology The Second Affiliated Hospital Zhejiang University School of Medicine Hangzhou China; ^2^ Department of Pathology Song‐Yang People's Hospital Lishui China; ^3^ Department of Gastroenterology Longquan People's Hospital Lishui China; ^4^ Department of Gastroenterology The Fourth Affiliated Hospital Zhejiang University School of Medicine Jinhua China

**Keywords:** biopsy site, case‐control study, *Helicobacter pylori* density, inflammation severity, inflammatory activity

## Abstract

**Background:**

While the Updated Sydney System classifies *Helicobacter pylori* density together with inflammatory scores, the clinical relevance of bacterial load remains uncertain. This study aimed to assess the association between histological *H. pylori* density and the severity of gastric inflammatory activity.

**Methods:**

We performed a retrospective analysis of 1680 treatment‐naive patients from a Chinese cohort (2022–2023). *H. pylori* density was categorized as low or high grades, with marked neutrophilic infiltration as the primary outcome. Multivariable logistic regression adjusted for potential confounders.

**Results:**

Among 2298 biopsy sites from 1680 patients (median age 52 years; 48.2% female), high *H. pylori* density showed no significant overall association with marked inflammation (all *p* > 0.050). While a significant inverse association was observed in non‐atrophic antral single‐site biopsies (adjusted odds ratio 0.52; 95% confidence interval 0.28–0.93), it was not reproduced in multi‐site analyses. Increasing age was associated with a lower prevalence of severe antral inflammation.

**Conclusions:**

Histological *H. pylori* density might therefore not be a reliable marker of neutrophilic activity, although a significant inverse association observed in a single‐site subgroup was not reproducible and may reflect sampling variability.

**Trial Registration:**

N/A

**Abbreviations**
aOR: adjusted odds ratio;
CI: confidence interval;
GEE: generalized estimating equations;
*H. pylori*
: *Helicobacter pylori*;
OR: odds ratio;
PPIs: proton pump inhibitors;
SD: standard deviation.

## 1 Introduction

The Updated Sydney System requires grading *Helicobacter pylori* density and inflammatory activity to standardize the histopathological diagnosis of gastritis [1]. Clinically, the prognostic significance of density remains controversial. One study identified a high *H. pylori* load as a risk factor for eradication resistance, while another found severe mucosal atrophy to be the primary determinant of treatment failure [2, 3]. Clinical reliance on density grading assumes that *H. pylori* load reflects disease severity, but this quantitative relationship has not been rigorously validated. Although the Maastricht VI/Florence consensus establishes *H. pylori* as the principal cause of gastritis [4], the correlation between bacterial burden and inflammatory activity severity is unclear. Environmental factors, including proton pump inhibitors (PPIs), can suppress histological density without reducing inflammation, indicating that colonization density may not linearly correlate linearly with inflammatory severity [5]. Nevertheless, this relationship is still understudied in treatment‐naïve populations.

Biologically, an inverse association is plausible. Severe inflammation may create a hostile microenvironment that limits *H. pylori* colonization [6], or low‐density states may fail to trigger regulatory T‐cell suppression, resulting in dysregulated innate immune responses characterized by intense neutrophilic infiltration [7, 8, 9, 10]. Furthermore, progressive atrophic gastritis inherently reduces the gastric niche suitable for *H. pylori* survival, potentially confounding density scores [11, 12]. Despite these complexities, previous epidemiological studies have often relied on single‐site biopsies, which overlook sampling variability and fail to adjust for co‐existing atrophy, intestinal metaplasia, and demographic confounders [13, 14].

This case‐control study evaluated the independent association between histological *H. pylori* density and the severity of gastric inflammatory activity. To address the methodological limitations of previous research, this study accounted for anatomical sampling variability (single vs. multiple sites), mucosal atrophy, and intestinal metaplasia in a treatment‐naïve population.

## 2 Materials and Methods

### 2.1 Ethical Considerations

This was a retrospective study using data from a case database; therefore, informed consent from participants was not required and was approved by the Institutional Review Board of Songyang People's Hospital (Approval Number: 20240814001,  Supporting information 3). Informed consent was waived due to the study's retrospective design. This study followed the Strengthening the Reporting of Observational Studies in Epidemiology reporting guideline.

### 2.2 Study Design, Setting, and Participants

A retrospective, hospital‐based, case‐control study used data from patients who underwent diagnostic upper endoscopy with gastric biopsy at Songyang People's Hospital from January 2022 to December 2023. The exposure was on the density of *H. pylori*. The outcome was the severity of inflammation activity, specifically neutrophilic infiltration. Unless otherwise stated, each biopsy site served as the unit of analysis. Case sites were defined as locations exhibiting severe histopathologic inflammation, while control sites were defined as locations with mild or moderate histopathologic inflammation. For simplicity, the grades of “none” and “mild” activity were combined and classified as “mild.” The presence of gastric atrophy and intestinal metaplasia at each site was noted as a confounding factor for subgroup analyses. All gastric biopsy samples were prepared for histopathological evaluation. *H. pylori* density was assessed histologically by methylene blue–boric acid staining. All slides were independently evaluated by two pathologists. In cases of disagreement, the slides were further reviewed by a third pathologist, whose assessment was considered final. The discordance rate between the initial two pathologists was approximately 7%. The severity of *H. pylori* density, inflammation activity, glandular atrophy, and intestinal metaplasia was graded using the Updated Sydney System [1]. The analysis was completed between January 2025 and January 2026.

### 2.3 Eligibility Criteria

The exclusion criteria for patients include: (1) any history of *H. pylori* eradication therapy, or use of antibiotics related to eradication therapy, PPIs, or potassium‐competitive acid blockers within 2 weeks before endoscopy; (2) active peptic ulcers with complications like bleeding, perforation, or obstruction; (3) a history of esophagectomy or gastrectomy; (4) a known allergy to the treatment drugs; (5) pregnancy or breastfeeding; (6) severe underlying conditions such as liver failure (aspartate aminotransferase or alanine aminotransferase greater than three times the upper limit of normal), kidney failure (creatinine of 2.0 mg/dL or higher or estimated glomerular filtration rate below 50 mL/min/1.73m^2^), immunodeficiency, cancer, or severe coronary artery disease like angina pectoris or stenosis of 75% or more; (7) missing relevant baseline data.

### 2.4 Statistical Analysis

All analyses were conducted at the biopsy site level. Continuous variables are reported as mean (standard deviation [SD]), and categorical variables as number (%). The association between *H. pylori* density and inflammation was evaluated using Spearman's rank correlation and Kendall's tau coefficients, with bootstrap resampling (10,000 iterations) to verify robustness. For age‐outcome associations, inverse probability of treatment weighting based on generalized propensity scores was employed, with weight quality assessed using effective sample size (>80%) and coefficient of variation (<0.5). Firth's penalized logistic regression was used when there was sparse data or quasi‐complete separation [15]. A two‐tailed *p*‐value of less than 0.05 was considered statistically significant. All analyses were calculated using R software, version 4.1.2.

## 3 Result

### 3.1 Study Characteristics

**FIGURE 1:**
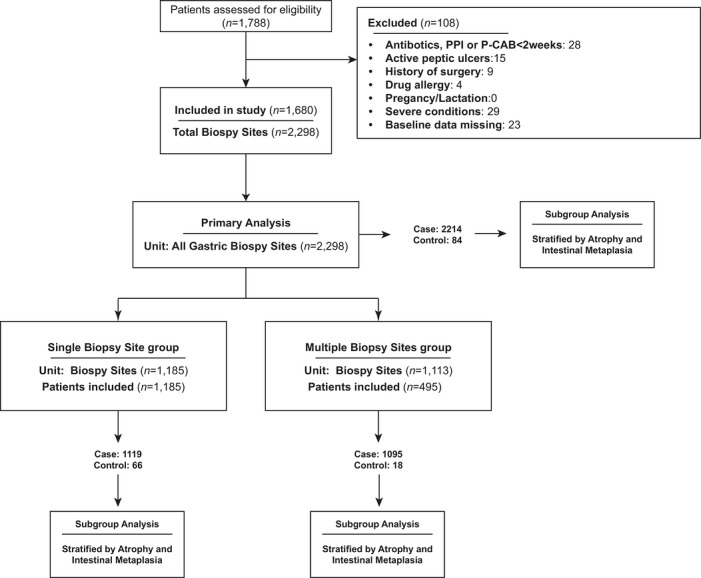
Patient enrollment flowchart. This flowchart illustrates the patient selection, exclusion, and grouping process for the retrospective case‐control study. Case group (with severe histopathologic inflammation), Control group (with mild or moderate histopathologic inflammation).

The study included 1680 treatment‐naïve patients with confirmed *H. pylori* infection, contributing 2298 gastric biopsy sites (Figure 1). Of these, 1185 patients provided a single biopsy site, while 495 provided multiple sites (totaling 1113 gastric biopsy sites). Baseline characteristics of the overall study population and of the case and control groups are shown in Table 1 and Table S1. A total of 2298 biopsy sites were included, comprising 84 case sites with severe inflammatory activity and 2214 control sites with mild or moderate inflammatory activity. Overall, biopsy sites in the case group were from older patients than those in the control group (median age, 57.56 vs. 51.85 years; *p* = 0.003). Gender distribution did not differ significantly between groups (*p* = 0.199), and no significant between‐group differences were observed for atrophy, intestinal metaplasia, or *H. pylori* density in the overall analysis (all *p* > 0.050). Similarly, age remained higher in the case group for antral biopsy sites (median age, 56.00 vs. 50.33 years; *p* = 0.019), whereas the difference was not statistically significant for incisura sites (*p* = 0.812). In the corpus and fundus subgroup, only 1 biopsy site was classified as a case. The corresponding baseline characteristics in the single–biopsy‐site and multiple–biopsy‐site subgroups are provided in Supporting Information 2.

**TABLE 1:** Baseline characteristics of the overall study population and the case and control groups.

	Inflammatory activity			
Characteristic ^a^, ^c^	**Overall, *n* (%)**	**Control, *n* (%)** ^b^	**Case, *n* (%)** ^b^	***p*** ^c^
Number of biopsy sites	2298	2214	84	
Age, median (IQR)	51.69 (13.07)	51.85 (13.00)	47.56 (14.24)	0.003
Gender				0.199
Male	1211 (52.7)	1173 (53.0)	38 (45.2)	
Female	1087 (47.3)	1041 (47.0)	46 (54.8)	
Atrophy				0.265
None	1917 (83.4)	1841 (83.2)	76 (90.5)	
Mild	262 (11.4)	255 (11.5)	7 (8.3)	
Moderate	100 (4.4)	99 (4.5)	1 (1.2)	
Severe	19 (0.8)	19 (0.9)	0 (0.0)	
Intestinal metaplasia				0.601
None	1642 (71.5)	1582 (71.5)	60 (71.4)	
Mild	513 (22.3)	492 (22.2)	21 (25.0)	
Moderate	107 (4.7)	104 (4.7)	3 (3.6)	
Severe	36 (1.6)	36 (1.6)	0 (0.0)	

**Abbreviation**: IQR, interquartile range.

^a^ All analyses were performed on a per‐biopsy‐site basis.

^b^ Case group (with severe histopathologic inflammation), Control group (with mild or moderate histopathologic inflammation).

^c^ Data are presented as median (IQR) for continuous variables and No. (%) for categorical variables. *p*‐Values were calculated for comparisons between the case and control groups within each biopsy‐site category.

### 3.2 Association of *H. pylori* Density with Activity Severity

**FIGURE 2:**
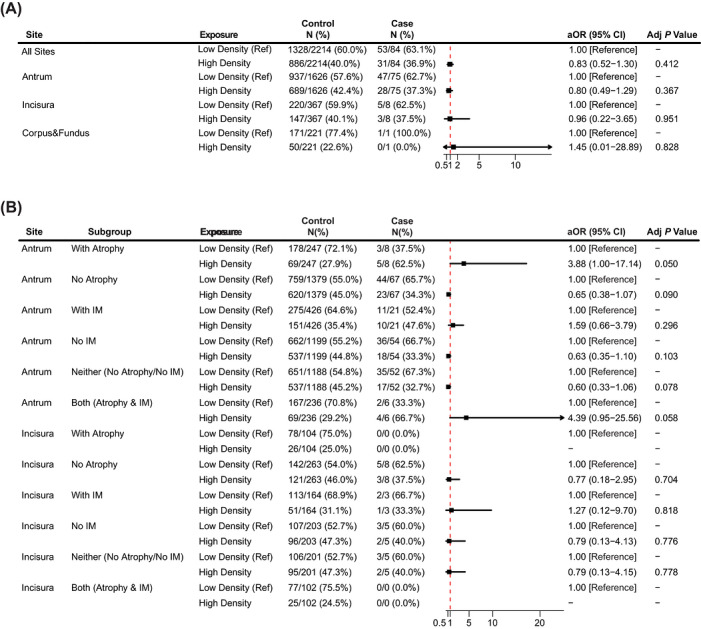
Association between *H. pylori* density and marked inflammatory activity across gastric biopsy sites in the overall population. (A) The association between *H. pylori* density and marked inflammatory activity in the overall population, including the all‐site analysis and site‐specific analyses for the antrum, incisura, and corpus/fundus. The all‐site analysis included biopsy specimens from the antrum, incisura, and corpus/fundus. (B) Subgroup analyses stratified by gastric atrophy and intestinal metaplasia within each biopsy site. Adjusted odds ratios (aORs) and 95% confidence intervals (CIs) were estimated using multivariable Firth's penalized logistic regression models. High *H. pylori* density indicates moderate to severe density, and low density indicates mild density. Case group (with severe histopathologic inflammation), Control group (with mild or moderate histopathologic inflammation). The vertical dashed line indicates no association (OR = 1.0). IM indicates intestinal metaplasia. Adj, adjusted; CI, confidence interval; *H. pylori*, *Helicobacter pylori*.

In the primary analysis, there was no correlation between *H. pylori* density and the degree of inflammatory activity, regardless of whether in the antrum or in the incisura (both *p* > 0.05; detailed in Table S2). After adjusting for age, gender, atrophy, and intestinal metaplasia, high *H. pylori* density was also not significantly associated with severe inflammatory activity at any biopsy site. High *H. pylori* density might not be associated with marked inflammatory activity in the all‐site analysis, including the antrum, incisura, and corpus/fundus (adjusted odds ratio [aOR], 0.83; 95% confidence interval [CI], 0.52–1.30; *p* = 0.412). The corresponding site‐specific aORs were 0.80 (95% CI, 0.49–1.29; *p* = 0.367) for the antrum, 0.96 (95% CI, 0.22–3.65; *p* = 0.951) for the incisura, and 1.45 (95% CI, 0.01–28.89; *p* = 0.828) for the corpus and fundus (Figure 2A). Subgroup analyses stratified by atrophy and intestinal metaplasia showed generally consistent results, with no statistically significant associations observed (Figure 2B).

### 3.3 Sensitivity Analyses by Sampling Strategy

**FIGURE 3:**
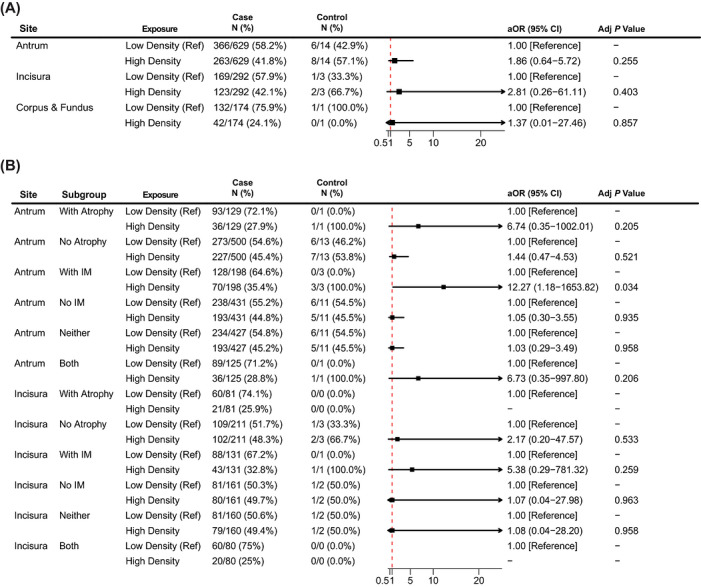
Subgroup analysis for *H. pylori* density and inflammation activity in patients with a multi‐site biopsy. (A) The association between *H. pylori* density (high vs low) and marked inflammatory activity across gastric biopsy sites (antrum, incisura, and corpus or fundus) in the multiple biopsy sites group. (B) Subgroup analyses stratified by the presence or absence of gastric atrophy and intestinal metaplasia for both antral and incisura biopsy sites. High *H. pylori* density indicates moderate to severe density, and low density indicates mild density. Case group (with severe histopathologic inflammation), Control group (with mild or moderate histopathologic inflammation). IM indicates intestinal metaplasia. aOR, adjusted odds ratio; CI, confidence interval; *H. pylori*, *Helicobacter pylori*.

To assess potential sampling bias, we compared the single‐biopsy‐site group with the multiple‐biopsy‐site group. Overall, the single‐biopsy‐site group comprised 66 case sites (5.6%) and 1119 control sites (94.4%), whereas the multiple‐biopsy‐site group comprised 18 case sites (1.6%) and 1095 control sites (98.4%). Among the 1185 patients with a single biopsy, high *H. pylori* density was not significantly associated with marked activity overall (antrum: aOR, 0.65 [95% CI, 0.37–1.11]; incisura: aOR, 0.97 [95% CI, 0.05–3.42]). However, in non‐atrophic antral biopsies, an inverse association was observed (aOR, 0.52 [95% CI, 0.28–0.93]; Table 2). This inverse association was not replicated in the multiple‐sites group (aOR, 1.44 [95% CI, 0.47–4.53]; Figure 3), suggesting potential sampling bias.

**TABLE 2:** Subgroup analysis for *H. pylori* density and inflammation activity in patients with a single‐site biopsy.

Characteristic	Adjusted OR (95% CI) ^a^			
*H. pylori* density	Overall	Without atrophy or IM	Without atrophy	Without IM
Antrum				
mild	[Reference]	[Reference]	[Reference]	[Reference]
moderate and Severe	0.65 (0.37, 1.11)	0.51 (0.25, 0.98)	0.52 (0.28, 0.93)	0.55 (0.27, 1.04)
Incisura				
mild	[Reference]	[Reference]	[Reference]	[Reference]
moderate and Severe	0.97 (0.05, 3.42)	0.72 (0.06, 5.93)	0.50 (0.05, 3.04)	0.54 (0.27, 1.02)

**Abbreviations**: CI, confidence interval; *H. pylori*, *Helicobacter pylori*; IM, Intestinal Metaplasia; OR, odds ratio.

^a^ Adjusted by age and gender. Odds ratios were estimated using Firth penalized likelihood logistic regression models. Case group (with severe histopathologic inflammation), Control group (with mild or moderate histopathologic inflammation).

### 3.4 Exploratory Analysis of Confounding Factors

To evaluate potential confounders of inflammatory activity, we examined the effects of gender and age. After propensity score matching (Figures S1), gender was not significantly associated with inflammation severity at any site (Table S3). Advanced age, however, was significantly associated with reduced severe antral inflammation (per 1‐SD increase: aOR, 0.74; 95% CI, 0.59–0.92; *p* = 0.008; Table S4).

## 4 Discussion

In this case‐control study of 1680 treatment‐naïve patients, we did not find evidence of an independent association between histological *H. pylori* density and the severity of neutrophilic infiltration in the overall population. Stratification by sampling strategy revealed a divergence. An inverse association was observed in single‐site antral biopsies, where high density correlated with reduced odds of marked inflammation; however, this association was not replicated in patients with multiple biopsy sites. These findings challenge the clinical utility of grading *H. pylori* load as an indicator for inflammatory activity and highlight the impact of sampling variability on histopathological assessment.

Our results differed from those of previous studies, which reported a positive density‐activity association [13, 14]. This discrepancy likely stems from statistical limitations in earlier analyses, such as quasi‐complete separation [16, 17]. Re‐analysis of data from Souissi et al., using Firth penalized regression to address sparse‐data bias, rendered the association non‐significant (*p* = 0.09) [15]. While these robust statistical methods address estimation bias, they cannot resolve the limitations inherent to retrospective data collection.

The inverse association observed in the single‐site non‐atrophic subgroup is likely a methodological artifact driven by sampling and selection biases rather than a true biological response. Given the well‐recognized patchy distribution of *H. pylori* colonization and neutrophilic activity within the gastric mucosa, single‐site biopsy sampling may generate spurious or non‐reproducible associations. This interpretation is further supported by the lack of a consistently significant association in advanced histological stages, where mucosal destruction would be expected to more uniformly influence bacterial colonization if a true biological mechanism existed [18]. Instead, the observed finding is more plausibly explained by information bias inherent to retrospective data, in which biopsy sites with normal or mild inflammation are under‐recorded relative to those with more pronounced pathology. This distortion may be further amplified by population‐level selection bias, as patients with mild symptoms are less likely to undergo endoscopy [19].

Although molecular genotyping was not performed, the discordance between density and inflammation likely reflects strain‐specific heterogeneity in virulence. Virulent strains expressing East Asian induce robust pro‐inflammatory signaling, driven by structural variations in the Glu‐Pro‐Ile‐Tyr‐Ala motifs that amplify Rat Sarcoma–Extracellular Signal–Regulated Kinase signaling pathway activation [20, 21, 22]. Meanwhile, the delivery of bacterial metabolites via the type IV secretion system triggers Alpha Kinase 1‐TRAF‐interacting protein with forkhead‐associated domain‐dependent nuclear factor‐kappa B signaling [23]. This immune response may accelerate *H. pylori* clearance, presenting as severe gastritis paradoxically accompanied by low *H. pylori* density. In contrast, regional analyses highlighted the presence of the less cytotoxic Vacuolating cytotoxin A middle region type 2 allele (17.6%‐56.4%), which may achieve high‐density colonization without marked inflammation [24, 25]. This phenotype is related to immune tolerance mechanisms mediated by regulatory T cells [7, 26, 27, 28] or lipid A modifications that evade Toll‐like receptor recognition [29, 30].

We acknowledge several limitations. First, the retrospective design precludes causal inference regarding the temporal progression of inflammatory activity. Longitudinal studies would be required to delineate the dynamic evolution of the density‐inflammation relationship.

Second, molecular genotyping was not performed, leaving the status of virulence factors such as cytotoxin‐associated gene A and VacA unknown. Although strain‐specific toxicity likely contributes to the observed discordance between *H. pylori* load and immune response, we were unable to adjust for these biological confounders or stratify analyses by virulence phenotypes.

Third, in the multiple‐biopsy group, we treated each biopsy specimen as an independent observation, without using generalized estimating equations (GEE) or mixed‐effects models to account for clustering within individuals. Since multiple samples from the same patient share identical host‐level covariates (e.g., age, gender, and genetic background), this approach violates the independence assumption of standard logistic regression. This may have resulted in underestimated standard errors for these patient‐level predictors, potentially leading to inflated Type I error rates and overstating the precision of our estimates [31].

Fourth, this study was conducted at a single center in China. The findings reflect a specific epidemiological context and may not be directly applicable to populations with different host genetic backgrounds or bacterial strain distributions. Therefore, extrapolation to Western cohorts or regions with low prevalence should be cautious, and validation in diverse settings is warranted.

Fifth, both *H. pylori* density and neutrophilic inflammatory activity were assessed using the Updated Sydney System on the same biopsy specimens. Because these parameters were evaluated within the same histological field, measurement coupling cannot be excluded, which may have limited their independence and influenced the observed association. Future studies using spatially distinct sampling, digital pathology–based quantification, or hierarchical measurement models may help better disentangle these correlated histological features.

Finally, although the study was derived from a relatively large histopathological dataset, the clinical relevance of the investigated association is limited. The relationship between *H. pylori* density and neutrophilic inflammatory activity does not directly inform key clinical outcomes such as gastric cancer risk, progression of mucosal atrophy, or eradication failure. Therefore, our findings should be interpreted primarily as a descriptive and methodological exploration within the Updated Sydney System. Future studies should focus on integrating density with clinically meaningful endpoints to better define its translational value.

In conclusion, in this retrospective case‐control study, the density of *H. pylori* is not significantly associated with the level of inflammatory activity in gastric biopsy sites across the overall population. This lack of connection was consistent in both primary and sensitivity analyses, including those at single‐site and multiple‐site biopsies. More evaluations that consider virulence factors and multi‐site biopsy strategies may provide a more reliable assessment of inflammation risk in people infected with *H. pylori*.

## Author Contributions

**Zhengchen Yu** and **Guochun Lou** take responsibility for the data source and the accuracy of the modeling analysis. **Zhengchen Yu** and **YongWei Wang** contributed to the study design and the model development. **Zhengchen Yu** and **BingQiang Xu** contributed to the literature search, data analysis, and expert interview. **Zhengchen Yu**, **Qin Du**, and **BingQiang Xu** contributed to the drafting of the manuscript. **Guochun Lou** and **Jun Ye** contributed to the critical revision of the manuscript.

## Funding

This work was supported by grants from the National Natural Science Foundation of China (No. 81773065) and the Natural Science Foundation of Zhejiang Province (No. LY21H160023).

## Ethics Statement

This study was reviewed and approved by the Ethics Committee of Songyang People's Hospital (approval No. 20240814001; August 4, 2024).

## Conflicts of Interest

The authors declare no conflicts of interest.

## Consent

N/A.

## Supporting information




**Supporting File 1**:
**Table S1**: Baseline characteristics of the case and control groups in the site‐specific subgroup.
**Table S2**: Correlation analysis between *H. pylori* density and inflammatory activity degree by anatomic site.
**Table S3**: Association of gender with the risk of severe inflammatory activity before and after propensity score matching stratified by anatomic site.
**Table S4**: Association of age with the risk of severe inflammation activity before and after generalized propensity score weighting stratified by anatomic site.

**Supporting File 2**: Baseline characteristics of patients in the single–biopsy‐site and multiple–biopsy‐site subgroups.

**Supporting File 3**: Institutional Review Board approval of the research protocol (Songyang People's Hospital).

**Supporting File 4**:
**Figure S1**: Covariate balance before and after propensity score matching for the analysis of the association between gender and severe inflammation activity.
**Note**: The Love plots illustrate the absolute standardized mean differences (SMDs) for baseline covariates between gender groups (female vs. male) before (red circles) and after (blue triangles) propensity score matching. The vertical dashed line represents the threshold for optimal balance (SMD < 0.1). (A) Results for the antrum. (B) Results for the incisura. High *H. pylori* density indicates moderate to severe density, and low density indicates mild density. Case group (with severe histopathologic inflammation), Control group (with mild or moderate histopathologic inflammation). Covariates adjusted in the propensity score model included age, *H. pylori* density, degree of atrophy, and degree of intestinal metaplasia.

## Data Availability

Data available on request due to restrictions, e.g., privacy or ethical restrictions. The data presented in this study are available upon request from the corresponding author. The data are not publicly available due to participant privacy.
