# Supplementary material for: Histological Helicobacter pylori Density Might Not be Associated With the Severity of Neutrophilic Inflammatory Activity
Source: DEN Open. 2026 May 27;7(1):e70356. doi: 10.1002/deo2.70356 (PMC13240408; doi:10.1002/deo2.70356)
Supplement: Supplementary file 1 — Supporting File 1: Table S1: Baseline characteristics of the case and control groups in the site‐specific subgroup. Table S2: Correlation analysis between H. pylori density and inflammatory activity degree by anatomic site. Table S3: Association of gender with the risk of severe inflammatory activity before and after propensity score matching stratified by anatomic site. Table S4: Association of age with the risk of severe inflammation activity before and after generalized propensity score weighting stratified by anatomic site. [file DEO2-7-e70356-s001.docx]

**Supporting information legend**

**Table S1.** Baseline Characteristics of the Case and Control Groups in the site-specific subgroup.

|  | **Inflammatory activity** | | |  |
| --- | --- | --- | --- | --- |
| **Site-specific subgroup**^1, 3^ | **Overall, n (%)** | **Control, n (%)** ^2^ | **Case, n (%)** ^2^ | ***P*** ^3^ |
| **Antrum** |  |  |  |  |
| Number of biopsy site | 1701 | 1626 | 75 |  |
| Age, median (IQR) | 50.17 (13.29) | 50.33 (13.21) | 46.67 (14.44) | 0.019 |
| Gender |  |  |  | 0.335 |
| Male | 875 (51.4) | 841 (51.7) | 34 (45.3) |  |
| Female | 826 (48.6) | 785 (48.3) | 41 (54.7) |  |
| Atrophy | 1446 (85.0) |  |  | 0.579 |
| None | 182 (10.7) | 1379 (84.8) | 67 (89.3) |  |
| Mild | 62 (3.6) | 175 (10.8) | 7 (9.3) |  |
| Moderate | 11 (0.6) | 61 (3.8) | 1 (1.3) |  |
| Severe | 1446 (85.0) | 11 (0.7) | 0 (0.0) |  |
| Intestinal Metaplasia | 1253 (73.7) |  |  | 0.688 |
| None | 357 (21.0) | 1199 (73.7) | 54 (72.0) |  |
| Mild | 67 (3.9) | 339 (20.8) | 18 (24.0) |  |
| Moderate | 24 (1.4) | 64 (3.9) | 3 (4.0) |  |
| Severe | 1253 (73.7) | 24 (1.5) | 0 (0.0) |  |
| **Incisura** |  |  |  |  |
| Number of biopsy site | 375 | 367 | 8 |  |
| Age, median (IQR) | 55.85 (11.62) | 55.87 (11.65) | 54.88 (11.00) | 0.812 |
| Gender |  |  |  |  |
| Male | 228 (60.8) | 224 (61.0) | 4 (50.0) | 0.790 |
| Female | 147 (39.2) | 143 (39.0) | 4 (50.0) |  |
| Atrophy |  |  |  | 0.371 |
| None | 271 (72.3) | 263 (71.7) | 8 (100.0) |  |
| Mild | 63 (16.8) | 63 (17.2) | 0 (0.0) |  |
| Moderate | 33 (8.8) | 33 (9.0) | 0 (0.0) |  |
| Severe | 8 (2.1) | 8 (2.2) | 0 (0.0) |  |
| Intestinal Metaplasia |  |  |  | 0.766 |
| None | 208 (55.5) | 203 (55.3) | 5 (62.5) |  |
| Mild | 121 (32.3) | 118 (32.2) | 3 (37.5) |  |
| Moderate | 35 (9.3) | 35 (9.5) | 0 (0.0) |  |
| Severe | 11 (2.9) | 11 (3.0) | 0 (0.0) |  |
| **Corpus & Fundus** |  |  |  |  |
| Number of biopsy site | 222 | 221 | 1 |  |
| Age, median (IQR) | 56.36 (10.98) | 56.36 (11.01) | 56.00 | **/** |
| Gender |  |  |  | 1.000 |
| Male | 108 (48.6) | 108 (48.9) | 0 (0.0) |  |
| Female | 114 (51.4) | 113 (51.1) | 1 (100.0) |  |
| Atrophy |  |  |  | 0.946 |
| None | 200 (90.1) | 199 (90.0) | 1 (100.0) |  |
| Mild | 17 (7.7) | 17 (7.7) | 0 (0.0) |  |
| Moderate | 5 (2.3) | 5 (2.3) | 0 (0.0) |  |
| Severe | 0 (0.0) | 0 (0.0) | 0 (0.0) |  |
| Intestinal Metaplasia |  |  |  | 0.973 |
| None | 181 (81.5) | 180 (81.4) | 1 (100.0) |  |
| Mild | 35 (15.8) | 35 (15.8) | 0 (0.0) |  |
| Moderate | 5 (2.3) | 5 (2.3) | 0 (0.0) |  |
| Severe | 1 (0.5) | 1 (0.5) | 0 (0.0) |  |

**Abbreviation:** IQR, interquartile range.

^1^ All analyses were performed on a per-biopsy-site basis.

^2^ Case group (with severe histopathologic inflammation), Control group (with mild or moderate histopathologic inflammation).

^3^ Data are presented as median (IQR) for continuous variables and No. (%) for categorical variables. *P*-values were calculated for comparisons between the case and control groups within each biopsy-site category.

**Table S2.** Correlation Analysis Between *H. pylori* Density and Inflammatory Activity Degree by Anatomic Site

|  | Antrum |  | Incisura |
| --- | --- | --- | --- |
| Spearman^1^ |  |  |  |
| ρ | 0.026 |  | 0.062 |
| *P*-value | 0.319 |  | 0.286 |
| Kendall ^2^ |  |  |  |
| τ | 0.025 |  | 0.060 |
| *P*-value | 0.320 |  | 0.284 |
| Bootstrap ^3^ |  |  |  |
| ρ | 0.026 |  | 0.526 |
| *P*-value | 0.519 |  | 0.528 |

^1^ Spearman’s rank correlation coefficient (ρ) was calculated to assess the strength and direction of the monotonic relationship between the ordinal variables.

^2^ Kendall’s tau (τ) statistic was used as a robust measure of ordinal association, accounting for tied ranks (duplicate scores) in the histological data.

^3^ Bootstrap Spearman correlation involved resampling the dataset 10,000 times to estimate the stability of the correlation coefficient and derive *P*-values based on the empirical distribution.

**Table S3.** Association of Gender With the Risk of Severe Inflammatory Activity Before and After Propensity Score Matching Stratified by Anatomic Site

|  | Sites in Antrum | | | |  | Sites in Incisura | | | |
| --- | --- | --- | --- | --- | --- | --- | --- | --- | --- |
| **Gender** | *N* (%) | Crude OR  (95% CI) | aOR  (95% CI) ^1^ | Adjusted *P*-value |  | *N* (%) | Crude OR  (95% CI) | aOR  (95% CI) ^1^ | Adjusted *P*-value |
| **Before PSM (875 vs. 826 in Antrum; 228 vs. 147 in Incisura)** | | | | | | |  |  |  |
| **Moderate-Severe** **Inflammation** |  |  |  | 0.216 |  |  |  |  | 0.683 |
| Female | 859 (98.17) | [Reference] | [Reference] |  |  | 226 (99.12) | [Reference] | [Reference] |  |
| Male | 803 (97.22) | 0.66 (0.34, 1.23) | 0.67 (0.35, 1.27) |  |  | 145 (98.64) | 0.64 (0.10, 4.20) | 0.73 (0.14, 3.26) |  |
| **Severe Inflammation** |  |  |  | 0.824 |  |  |  |  | 0.648 |
| Female | 34 (3.89) | [Reference] | [Reference] |  |  | 4 (1.75) | [Reference] | [Reference] |  |
| Male | 41 (4.96) | 1.29 (0.81, 2.06) | 0.88 (0.25, 2.75) |  |  | 4 (2.72) | 0.21 (0.02, 0.89) | 0.67 (0.11, 4.30) |  |
| **After PSM (804 vs. 804 in Antrum; 142 vs. 142 in Incisura)** ^2^ | | | | | | |  |  |  |
| **Moderate-Severe Inflammation** |  |  |  | 0.441 |  |  |  |  | / |
| Female | 789 (98.13) | [Reference] | [Reference] |  |  | 140 (98.59) | [Reference] | [Reference] |  |
| Male | 789 (97.14) | 0.65 (0.34, 1.24) | 0.65 (0.34, 1.26) |  |  | 140 (98.59) | 1.00 (0.15, 6.55) | / |  |
| **Severe Inflammation** |  |  |  | 0.842 |  |  |  |  | / |
| Female | 33 (4.10) | Ref | [Reference] |  |  | 3 (2.11) | [Reference] | [Reference] |  |
| Male | 37 (4.60) | 1.56 (0.40, 6.15) | 0.88 (0.23, 3.15) |  |  | 4 (2.82) | 1.69 (0.41, 7.48) | / |  |

**Abbreviation:** aOR, adjusted odds ratio; CI, confidence interval; OR, odds ratio; PSM, propensity score matching.

^1^ The multivariable models were adjusted for age, *H. pylori* density, degree of atrophy, and degree of intestinal metaplasia. Case group (with severe histopathologic inflammation), Control group (with mild or moderate histopathologic inflammation).

^2^ Propensity score matching was performed using a 1:1 ratio with a caliper width of 0.2.

**Table S4.** Association of Age With the Risk of Severe Inflammation Activity Before and After Generalized Propensity Score Weighting Stratified by Anatomic Site.

|  | Sites in Antrum | | | | |  | Sites in Incisura | | | | |
| --- | --- | --- | --- | --- | --- | --- | --- | --- | --- | --- | --- |
|  | **Moderate-Severe Inflammation** | |  | **Severe Inflammation** | |  | **Moderate-Severe Inflammation** | |  | **Severe Inflammation** | |
| **Methods** | OR (95% CI) | *P*-value |  | OR (95% CI) | *P*-value |  | OR (95% CI) | *P*-value |  | OR (95% CI) | *P*-value |
| **Unadjusted** |  |  |  |  |  |  |  |  |  |  |  |
| per 1year | 0.99 (0.96, 1.01) |  |  | 0.98 (0.96, 1.00) |  |  | / |  |  | 0.99 (0.94, 1.05) |  |
| per SD year | 0.84 (0.61, 1.15) | 0.269 |  | 0.76 (0.61, 0.96) | 0.020 |  | / | / |  | 0.90 (0.47, 1.85) | 0.760 |
|  |  |  |  |  |  |  |  |  |  |  |  |
| **GPS** ^1^ |  |  |  |  |  |  |  |  |  |  |  |
| per 1year | 0.99 (0.96, 1.01) |  |  | 0.98 (0.96, 0.99) |  |  | / | / |  | 1.00 (0.94, 1.06) |  |
| per SD year | 0.83 (0.61, 1.14) | 0.259 |  | 0.74 (0.59, 0.92) | 0.008 |  | / | / |  | 0.94 (0.47, 2.04) | 0.871 |

**Abbreviation**: CI, confidence interval; CV, coefficient of variation; ESS, effective sample size; GPS, generalized propensity score; OR, odds ratio; SD, standard deviation.

**Note**: Age was analyzed as a continuous variable (SD = 13.3 years for antrum; SD = 11.6 years for incisura). The symbol "/" indicates insufficient events for estimation. GPS weight quality: Antrum ESS = 1623.6 (95.4%), CV = 0.218; Incisura ESS = 351.7 (93.8%), CV = 0.258.

^1^ GPS model adjusted for gender, *H. pylori* density, degree of atrophy, and degree of intestinal metaplasia. Firth's penalized likelihood logistic regression was used. Case group (with severe histopathologic inflammation), Control group (with mild or moderate histopathologic inflammation).

**Figure S1.** Covariate Balance Before and After Propensity Score Matching for the Analysis of the Association Between Gender and Severe Inflammation Activity.

**Note:** The Love plots illustrate the absolute standardized mean differences (SMDs) for baseline covariates between gender groups (female vs male) before (red circles) and after (blue triangles) propensity score matching. The vertical dashed line represents the threshold for optimal balance (SMD < 0.1). (A) Results for the antrum. (B) Results for the incisura. High *H. pylori* density indicates moderate to severe density, and low density indicates mild density. Case group (with severe histopathologic inflammation), Control group (with mild or moderate histopathologic inflammation). Covariates adjusted in the propensity score model included age, *H. pylori* density, degree of atrophy, and degree of intestinal metaplasia.
