# Supplementary material for: Histological Helicobacter pylori Density Might Not be Associated With the Severity of Neutrophilic Inflammatory Activity
Source: DEN Open. 2026 May 27;7(1):e70356. doi: 10.1002/deo2.70356 (PMC13240408; doi:10.1002/deo2.70356)
Supplement: Supplementary file 3 — Supporting File 3: Institutional Review Board approval of the research protocol (Songyang People's Hospital). [file DEO2-7-e70356-s002.pdf]

Certificate of Medical Ethics Review (Songyang People's Hospital)

Title	**The Relationship between Helicobacter pylori Abundance and Gastric Pathological Changes: A Retrospective Cohort Study Based on a Bayesian Multivariate Model**
First Author	Guochun Lou
Author Affiliation	Songyang Branch, The Second Affiliated Hospital of Zhejiang University School of Medicine
Materials reviewed by the Ethics Committee	☐Study protocol
	☐Informed consent form
	☑Other materials
Summary of the research involving human participants and study protocol	This retrospective cohort study was conducted in Zhejiang Province, China, from January 1, 2022, to December 31, 2023. The associations between H. pylori abundance and gastric pathological changes were assessed using a Bayesian multivariate model. Nonlinear correlations between patient age and gastric pathological changes were explored using restricted cubic spline (RCS) curve analysis.
Opinion of the Ethics Committee	Reviewed and approved.
Approval Number	20240814001
Ethics Committee	Ethics Committee of Songyang People's Hospital
Date	August 4, 2024
Seal & Signature	
