# Supplementary material for: Histological Helicobacter pylori Density Might Not be Associated With the Severity of Neutrophilic Inflammatory Activity
Source: DEN Open. 2026 May 27;7(1):e70356. doi: 10.1002/deo2.70356 (PMC13240408; doi:10.1002/deo2.70356)
Supplement: Supplementary file 4 — Supporting File 4: Figure S1: Covariate balance before and after propensity score matching for the analysis of the association between gender and severe inflammation activity. Note: The Love plots illustrate the absolute standardized mean differences (SMDs) for baseline covariates between gender groups (female vs. male) before (red circles) and after (blue triangles) propensity score matching. The vertical dashed line represents the threshold for optimal balance (SMD < 0.1). (A) Results for the antrum. (B) Results for the incisura. High H. pylori density indicates moderate to severe density, and low density indicates mild density. Case group (with severe histopathologic inflammation), Control group (with mild or moderate histopathologic inflammation). Covariates adjusted in the propensity score model included age, H. pylori density, degree of atrophy, and degree of intestinal metaplasia. [file DEO2-7-e70356-s004.pdf]

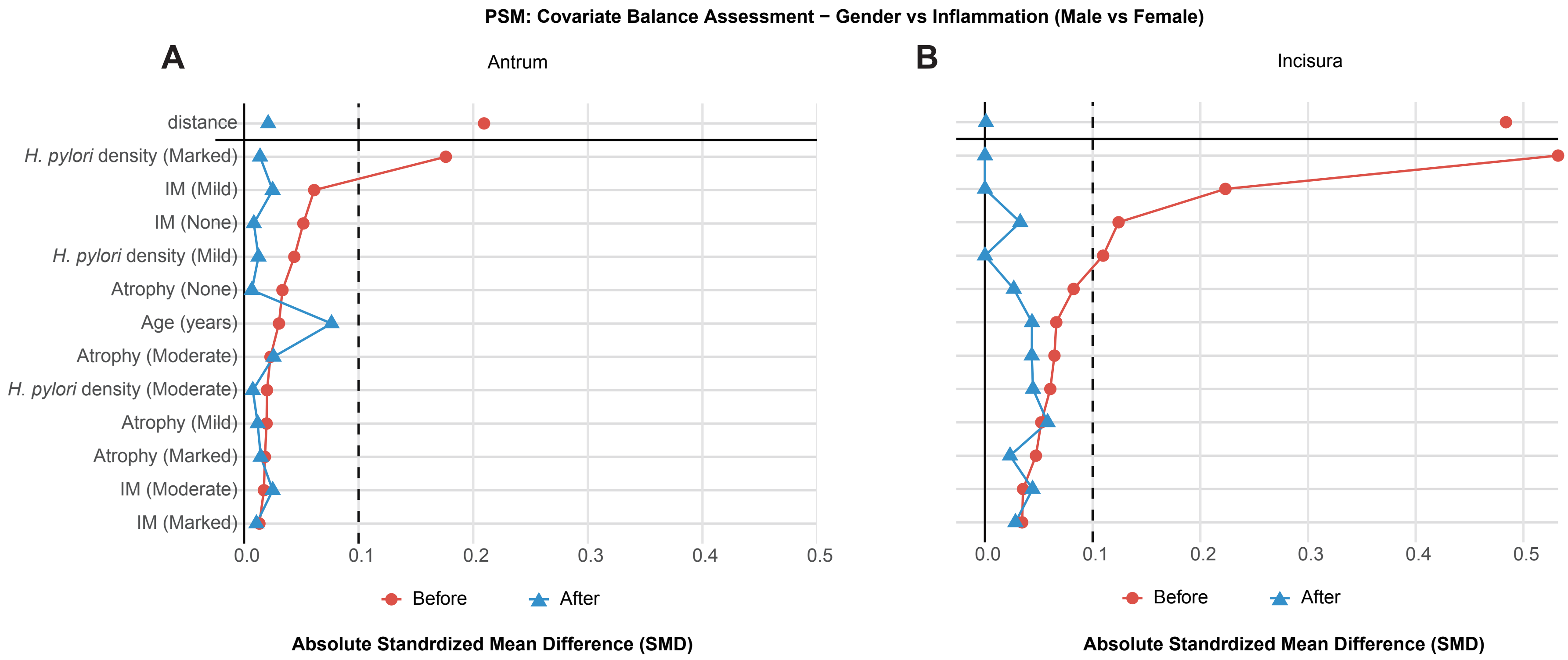

PSM: Covariate Balance Assessment − Gender vs Inflammation (Male vs Female)
A
Antrum
distance
H. pylori density (Marked)
IM (Mild)
IM (None)
H. pylori density (Mild)
Atrophy (None)
Age (years)
Atrophy (Moderate)
H. pylori density (Moderate)
Atrophy (Mild)
Atrophy (Marked)
IM (Moderate)
IM (Marked)
0.0
0.1
0.2
0.3
0.4
0.5
Before
After
Absolute Standrdized Mean Difference (SMD)
B
Incisura
0.0
0.1
0.2
0.3
0.4
0.5
Before
After
Absolute Standrdized Mean Difference (SMD)
